# B Lymphocyte-Deficiency in Mice Causes Vascular Dysfunction by Inducing Neutrophilia

**DOI:** 10.3390/biomedicines9111686

**Published:** 2021-11-14

**Authors:** Ning Xia, Solveig Hasselwander, Gisela Reifenberg, Alice Habermeier, Ellen I. Closs, Maximilian Mimmler, Rebecca Jung, Susanne Karbach, Jérémy Lagrange, Philip Wenzel, Andreas Daiber, Thomas Münzel, Nadine Hövelmeyer, Ari Waisman, Huige Li

**Affiliations:** 1 Department of Pharmacology, Johannes Gutenberg University Medical Center, 55131 Mainz, Germany; xianing@uni-mainz.de (N.X.); sohassel@uni-mainz.de (S.H.); reifenb@uni-mainz.de (G.R.); habermei@uni-mainz.de (A.H.); closs@uni-mainz.de (E.I.C.); mmimmler@students.uni-mainz.de (M.M.); 2 Institute for Molecular Medicine, Johannes Gutenberg University Medical Center, 55131 Mainz, Germany; rebeccajung@uni-mainz.de (R.J.); hoevelme@uni-mainz.de (N.H.); waisman@uni-mainz.de (A.W.); 3 Center for Thrombosis and Hemostasis, Johannes Gutenberg University Medical Center, 55131 Mainz, Germany; karbasu@uni-mainz.de (S.K.); jlagrang@uni-mainz.de (J.L.); wenzelp@uni-mainz.de (P.W.); 4 Department of Cardiology, Cardiology 1, Johannes Gutenberg University Medical Center, 55131 Mainz, Germany; daiber@uni-mainz.de (A.D.); tmuenzel@uni-mainz.de (T.M.); 5 German Center for Cardiovascular Research (DZHK), Partner Site Rhine-Main, 55131 Mainz, Germany; 6 Research Center for Immunotherapy (FZI), Johannes Gutenberg University Medical Center, 55131 Mainz, Germany

**Keywords:** B lymphocytes, vascular function, neutrophil granulocytes, nitric oxide

## Abstract

B lymphocytes have been implicated in the development of insulin resistance, atherosclerosis and certain types of hypertension. In contrast to these studies, which were performed under pathological conditions, the present study provides evidence for the protective effect of B lymphocytes in maintaining vascular homeostasis under physiological conditions. In young mice not exposed to any known risk factors, the lack of B cells led to massive endothelial dysfunction. The vascular dysfunction in B cell-deficient mice was associated with an increased number of neutrophils in the circulating blood. Neutrophil depletion in B cell-deficient mice resulted in the complete normalization of vascular function, indicating a causal role of neutrophilia. Moreover, vascular function in B cell-deficient mice could be restored by adoptive transfer of naive B-1 cells isolated from wild-type mice. Interestingly, B-1 cell transfer also reduced the number of neutrophils in the recipient mice, further supporting the involvement of neutrophils in the vascular pathology caused by B cell-deficiency. In conclusion, we report in the present study the hitherto undescribed role of B lymphocytes in regulating vascular function. B cell dysregulation may represent a crucial mechanism in vascular pathology.

## 1. Introduction

Recent studies have implicated B lymphocytes in the pathogenesis of insulin resistance, atherosclerosis and hypertension. It has been shown that B cells accumulate in the visceral adipose tissue of diet-induced obese mice. B cell-deficient mice on a high-fat diet are protected from insulin resistance despite weight gain. The pathogenic effects of B cells are attributed to activation of proinflammatory macrophages and T cells and to the production of IgG antibodies. Transfer of IgG from obese wild-type mice into B cell-deficient mice induces insulin resistance and glucose intolerance [1].

Both the proatherogenic and antiatherogenic effects of B cells have been shown in mouse models of atherosclerosis [2,3]. B cells are present in the adventitia and in the perivascular adipose tissue of both normal and atherosclerotic arteries [4]. This has been shown in mouse studies [4,5] as well as in human arteries [6,7]. Moreover, the number of B cells in the vascular wall increases in parallel with the severity of the atherosclerotic lesion. There are subset- and context-specific differences in B cell function in atherosclerosis in mice. While B-1 cells [8,9,10,11] have been shown to be atheroprotective by secreting natural IgM antibodies, innate response activator (IRA) B cells [12] are pro-atherogenic by promoting the expansion of classic dendritic cells, which then generate interferon (IFN)-γ-producing T helper (Th1) cells. Conventional B-2 cells [13] promote atherosclerosis by stimulating T cell and dendritic cell activation and Th1 polarization, as well as by producing pathogenic IgG antibodies against modified lipids, oxidized low-density lipoprotein (LDL) and malondialdehyde-LDL. Regulatory B cells (Breg) mainly suppress autoimmunity by producing interleukin-10 (IL-10) and through direct interaction with other leukocytes, but their role in atherosclerosis remains controversial [14,15,16,17].

B lymphocytes and other immune cells are also involved in hypertension [18,19,20]. Angiotensin II-induced hypertension is ameliorated in mice lacking both B and T lymphocytes [21] as well as in mice deficient solely in B cells [22]. Moreover, plasma cell depletion has been shown to attenuate hypertension in a mouse model of systemic lupus erythematosus, indicating a contribution of B cells to autoimmune-associated hypertension [23].

However, there is to date no study reporting the role of B lymphocytes in regulating vasomotor function. We show in the present study that B cell-deficiency in mice leads to a massive endothelial dysfunction and provide the first evidence of a hitherto unidentified role for B cells in maintaining vascular hemostasis under physiological conditions.

## 2. Methods

### 2.1. Animals

The wild-type C57BL/6J mice were from Janvier Labs (Le Genest-Saint-Isle, France). JHT mice were generated by the deletion of the exons encoding the joining region and the intron enhancer of the immunoglobulin heavy chain locus [24]. Homozygous JHT mice produce no functional B cells and secrete no antibodies [24]. IgMi mice were generated by deleting the first polyA site of the constant region coding for heavy chain of IgM essential for maturation of the RNA that is transcribed to the secreted form of the IgM heavy chain (secreted IgM antibodies). The second poly A site, which is responsible for the maturation of the membrane form of the IgM heavy chain (IgM B cell receptor) was left intact [25]. The resulting IgMi mice developed B cells expressing IgM at the surface but did not secret antibodies [26]. By contrast, IgG1i mice express membrane IgG1 as the B cell receptor and secrete IgG1, but no other antibody subtypes [26]. The genetic background of the all mouse strains used in the present study is C57BL/6J. The animal experiment was approved by the responsible regulatory authority (Landesuntersuchungsamt Rheinland-Pfalz; 23 177-07/G 12-1-021 & G 17-1-020) and was conducted in accordance with German animal protection law, the EU Directive 2010/63/EU for animal experiments and the National Institutes of Health (NIH) Guide for the Care and Use of Laboratory Animals.

### 2.2. Neutrophil Depletion

To deplete their neutrophils, JHT mice were treated with a single intraperitoneal injection of a neutrophil-specific anti-Ly6G antibody (0.5 mg/mouse; Clone 1A8; # BP0075-1, Bio-X-Cell, Lebanon, NH, USA). IgG2a served as an isotype control. The depletion of neutrophils occurred within 24 h and lasted for more than 3 days, as previously described [27]. A vascular function study was performed 36 h after anti-Ly6G antibody injection.

### 2.3. B Cell Transfer Experiments

The adoptive B cell transfer experiments were performed at the age of 8 weeks (both donor and recipient mice). At this age, the immune system of the mice is fully developed [28]. B-1 cells were isolated from the peritoneal cavity of the wild-type mice by negative selection with magnetic-activated cell sorting (MACS) cell separation [29]. First, B cells were isolated with the Pan B Cell Isolation Kit II (130-104-443, Miltenyi Biotec, Bergisch Gladbach, Germany). Next, B-2 cells were excluded using CD23 MicroMeads (130-098-784, Miltenyi Biotec) and peritoneal macrophages excluded with anti-F4/80 MicroBeads UltraPure (130-110-443, Miltenyi Biotec) to obtain the B-1 cells. The B-2 cells were isolated from the spleen of wild-type mice with negative selection strategy by excluding B-1 cells using CD43 MicroBeads (130-049-801, Miltenyi Biotec) from the B cells isolated with the Pan B Cell Isolation Kit II [30]. B cell-deficient JHT mice were transferred with 5 × 10^6^ B-1 or B-2 cells, respectively [30]. Two weeks after B cell transfer, the aortae were isolated from the recipient mice for the myograph experiments.

### 2.4. Myograph Experiments

For the vascular function studies, the thoracic aortae was dissected into rings 2–3 mm in length. Perivascular adipose and connective tissues were removed. Isometric tension was recorded using a wire myograph system (Danish Myo Technology, Aarhus, Denmark). The rings were equilibrated for 60 min and contracted twice with 120 mmol/L KCl. For the assessment of vascular function, the rings were pre-contracted with noradrenaline to reach submaximal tension (80% of that obtained with 120 mmol/L KCl), before vasodilatation was induced by acetylcholine [31,32].

### 2.5. Electron Paramagnetic Resonance (EPR)

The thoracic aorta samples were incubated for 30 min at 37 °C in a Krebs–Henseleit buffer supplemented with 200 µM colloid Fe(DETC)_2_, as described previously [33,34]. A table-top X-band spectrometer Miniscope MS400 (Magnettech, Berlin, Germany) was utilized for EPR performance and a Dewar flask (Wilmad, Buena, New Jersey) at 77 K for recordings.

### 2.6. Measurement of ROS Production

Aortic ROS production was measured with L-012 chemiluminescence [35]. The thoracic aortae were pre-incubated with 100 µM of the luminol derivate 8-amino-5-chloro-7-phenylpyridol [3,4-d]pyridazine-1,4-(2H,3H)dione sodium salt (L-012) and a chemiluminescence measurement was performed after a dark adaptation for 30 min [33].

### 2.7. Gene Expression Analyses

The RNA was isolated using peqGOLD TriFast™ (PEQLAB) and the cDNA was generated with the High Capacity cDNA Reverse Transcription Kit (Applied Biosystems, Waltham, MA, USA). Quantitative real time RT-PCR (qPCR) reactions were performed on a StepOnePlus™ Real-Time PCR System (Applied Biosystems) using SYBR^®^ Green JumpStart™ Taq ReadyMix™ (Sigma-Aldrich, Taufkirchen, Germany) and 20 ng of cDNA. The relative mRNA levels of the target genes were quantified using a comparative threshold C_T_ normalized to the housekeeping gene TATA-binding protein (TBP) [34]. The mRNA expression in the wild-type control mice was set to 100%. The qPCR primer sequences were as follows: eNOS_forward: CCT TCC GCT ACC AGC CAG A, eNOS_reverse: CAG AGA TCT TCA CTG CAT TGG CTA; SOD2_forward: GCT CTG GCC AAG GGA GAT G, SOD2_reverse: TGT CCC CCA TTG AAC TT; TBP_forward: CTT CGT GCA AGA AAT GCT GAA T, TBP_reverse: CAG TTG TCC GTG GCT CTC TTA TT [32].

### 2.8. Western Blot Analyses

Western blot analyses were performed with total protein samples (30 µg each) from the aorta. The following primary antibodies were used: mouse monoclonal antibody against eNOS (catalog number 610297, BD Transduction Laboratories, Franklin Lakes, NJ, USA; 1:2000), SOD2 (ADI-SOD-110, Enzo Life Sciences, New York, NY, USA; 1:2000). The Western blot was performed as previously described [33,34]. The protein samples were separated on a Bis-Tris gel and transferred to a nitrocellulose membrane. The blots were blocked in 5% milk powder in TBST (10 mM Tris-HCl, pH 7.4, 150 mM NaCl with 0.1% Tween 20) for one hour at room temperature. The primary antibodies were diluted in the same solution used for blocking at 4 °C overnight. The blots were then washed in TBST and incubated with a horseradish peroxidase-conjugated secondary antibody diluted in 5% milk in TBST for one hour. After washing in TBST and then in TBS, the immunocomplexes were visualized using an enhanced horseradish peroxidase/luminol chemiluminescence reagent (PerkinElmer Life and Analytical Sciences, Boston, MA, USA), according to the manufacturer’s instructions. A densitometric analysis of scanned blots was performed using Quantity One software (Bio-Rad, Feldkirchen, Germany).

### 2.9. Flow Cytometric Analyses

The aortae were cleaned of perivascular adipose tissue and digested with liberase (1 mg/mL; Roche Diagnostics, Basel, Switzerland), as described previously [36]. The single-cell suspensions were treated with Fc-block and washed. The cells were stained for 1 h with commercial antibodies. The samples were analyzed using a BD FACS CANTO II flow cytometer (Becton Dickinson, Franklin Lakes, NJ, USA) and FACSDiva Sofware 7.0 (Becton Dickinson), respectively. For details, see the online Supplementary Material.

### 2.10. Measurement of Neopterin in Mouse Serum

Neopterin was measured in the serum samples with high-performance liquid chromatography (HPLC), as previously described [37]. Briefly, 20 µL of serum was added with 80 µL 100% acetonitrile and placed on ice for 15 min to precipitate the proteins. The samples were then centrifuged (4 °C and 20,000× *g* for 15 min). A total of 15 µL of the supernatant was injected onto an XSelect CSH C18 3.5 µm column (Waters GmbH, Eschborn, Germany) with a mobile phase of 0.7% acetonitrile in a 20 mM phosphate buffer, pH 6.2. The eluted neopterin was detected by its native fluorescence at emission 438 nm, excitation 353 nm, using a Shimadzu RF-20A detector, and analyzed with McDAcq Software.

### 2.11. Statistical Analysis

The results are expressed as mean ± SD (standard deviation) or SEM (standard error of the mean). A two-tailed, unpaired Student’s *t*-test was used for the comparison of the two groups. Two-way analysis of variance (ANOVA) was performed to compare more than two groups or tow curves. The *p* values < 0.05 were considered significantly different. The statistical analysis was performed with GraphPad Prism 7.02 (GraphPad Software, La Jolla, CA, USA).

## 3. Results

### 3.1. B Cell-Deficiency in Mice Leads to Vascular Dysfunction and Reduced NO Production

**Figure 1:**
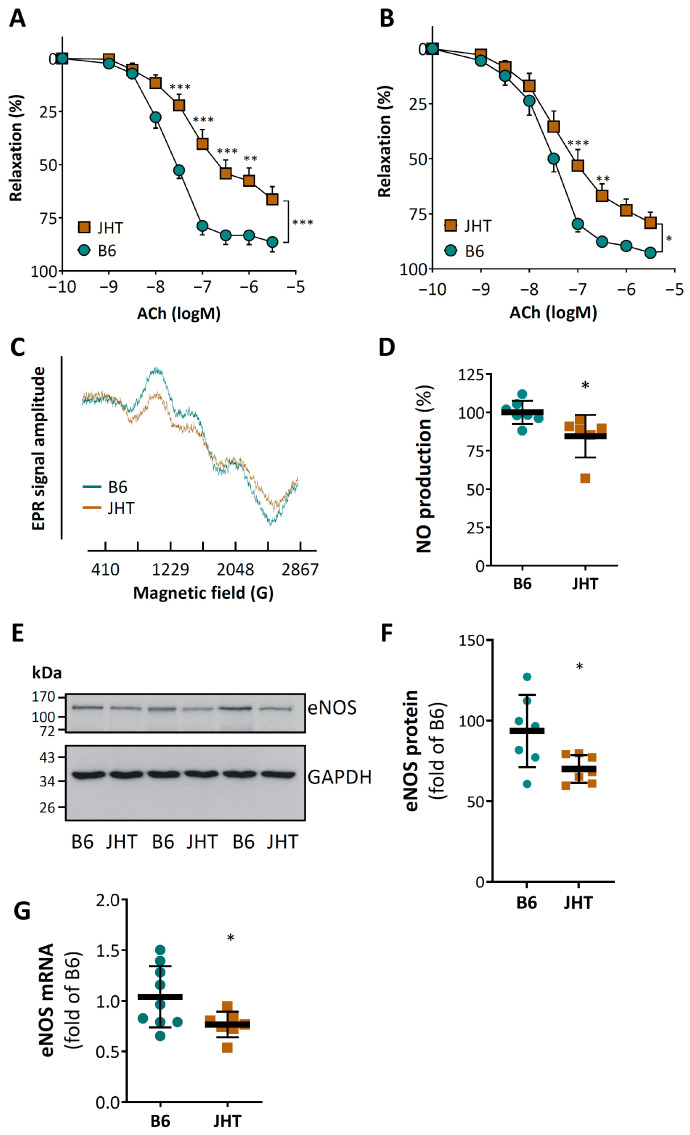
B cell-deficiency in mice leads to endothelial dysfunction and reduced NO production. Aortae were isolated from wild-type C57BL/6J and B cell-deficient JHT mice at the age of 17 (**A**) or 7 (**B**) weeks, respectively. Vasodilator function was studied using a wire myograph system. Aortic rings were precontracted with noradrenaline and vasodilation was induced with acetylcholine (ACh) at increasing concentrations. Symbols represent mean ± SEM. n = 5 (**A**) and 12 (**B**), respectively. NO production was determined by electron paramagnetic resonance (EPR) with 200 µM Fe(DETC)_2_ using aorta samples isolated from 17 week-old mice (**C**,**D**). The protein (**E**,**F**) and mRNA (**G**) expression of eNOS was analyzed with Western blotting and qPCR, respectively. The horizontal lines in the scatter dot plots represent the mean ± SD (**D**,**F**,**G**). * *p* < 0.05, ** *p* < 0.01, *** *p* < 0.001, compared with wild-type mice (B6); two-way ANOVA (**A**,**B**) and unpaired *t*-test (**D**,**F**,**G**), respectively.

The vasodilator response to acetylcholine was markedly reduced in aortic rings from the 17 week-old male JHT mice compared to the age-matched wild-type C57BL/6J mice (Figure 1A). This vascular dysfunction was also observed in younger mice at the age of 7 weeks (Figure 1B). No significant changes in blood pressure were found in the JHT mice at the age of 17 weeks (Supplementary Figure S1).

The vascular dysfunction of the JHT mice was associated with reduced vascular nitric oxide (NO) production (Figure 1C,D). The expression of endothelial NO synthase (eNOS) at both protein and mRNA levels in the aortae of the JHT mice was lower than that in the wild-type controls (Figure 1E–G). By contrast, no changes in the expression of neuronal (nNOS) or inducible NOS (iNOS) were found (Supplementary Figure S2).

### 3.2. B Cell-Deficiency in Mice Leads to Vascular Oxidative Stress and Inflammation

**Figure 2:**
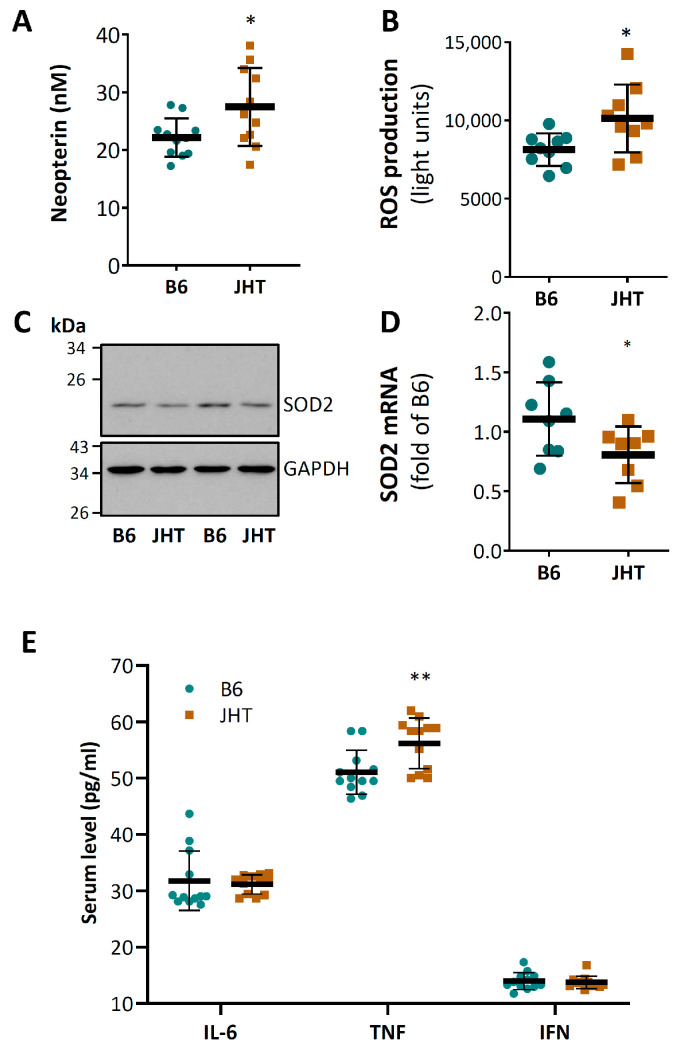
B cell-deficiency in mice leads to vascular oxidative stress and inflammation. Neopterin concentration in blood of 17-week-old mice was measured with HPLC (**A**). Aortic ROS production was determined with L-012 chemiluminescence (**B**). SOD2 protein expression (**C**) was analyzed with Western blotting. mRNA expression was studied by qPCR using aorta samples (**D**). The horizontal lines in the scatter dot plots (**A**,**B**,**D**,**E**) represent mean ± SD. * *p* < 0.05, ** *p* < 0.01, compared with wild-type mice (B6); unpaired *t*-test.

The JHT mice showed higher serum concentrations of neopterin (Figure 2A), a marker of oxidative stress and immune activation. In agreement with this, an increased vascular ROS level was observed in the aorta (Figure 2B). The mRNA and protein levels of SOD2 were reduced in the aortae of the JHT mice (Figure 2C,D). No significant expressional changes were observed for other antioxidant or prooxidant enzymes including SOD1, SOD3, GPx1, HO-1, catalase, NOX1, NOX2, p22phox, and p47phox (Supplementary Figure S3).

The ELISA measurements showed increased serum levels of TNF-α in the JHT mice, whereas no significant changes in the serum levels of IL-6 or interferon-γ (IFN-γ) were found (Figure 2E).

**Figure 3:**
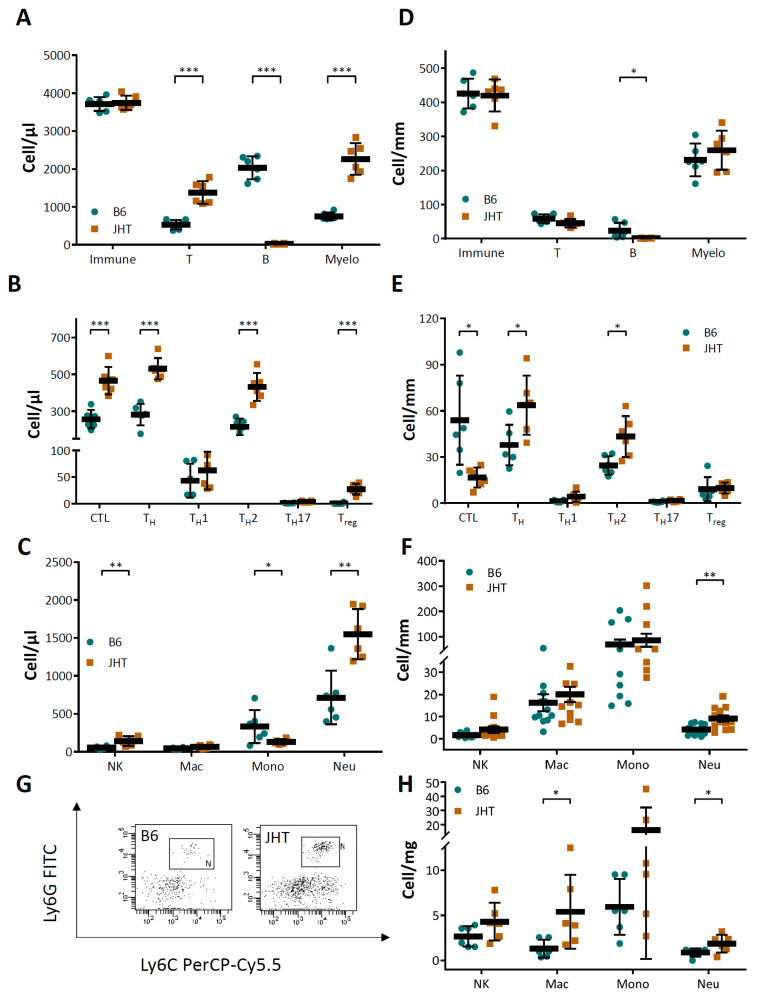
B cell-deficiency in mice leads to neutrophilia and neutrophil infiltration in aortic tissues. Immune cell profile was analyzed with FACS using blood (**A**–**C**), aorta (**D**–**F**), and aortic PVAT (**G**,**H**) samples from 17-week-old wild-type C57BL/6J and B cell-deficient JHT mice, respectively. The horizontal lines in the scatter dot plots represent mean ± SD. * *p* < 0.05, ** *p* < 0.01, *** *p* < 0.001; unpaired *t*-test.

The JHT mice demonstrated increased numbers of T cells and myelomonocytic cells in the circulating blood (Figure 3A). The increase in T cell frequencies was reflected in the T cell cytotoxic T lymphocytes (CTL), Th2, as well as the regulatory T cells (Treg) (Figure 3B). The increase in the circulating myelomonocytic cells was mainly caused by an elevation in the neutrophil numbers (Figure 3C). Increased numbers of T cells and neutrophils were also observed in the aortae (Figure 3D–F) as well as in aortic PVAT (Figure 3G,H). Neutrophil proteins, including myeloperoxidase, neutrophil elastase, and metalloproteinase 9, were also found increased in the aortae or aortic PVAT (Supplementary Figures S4–S6).

### 3.3. Vascular Dysfunction in B Cell-Deficiency in Mice Is Correlated with Neutrophilia

**Figure 4:**
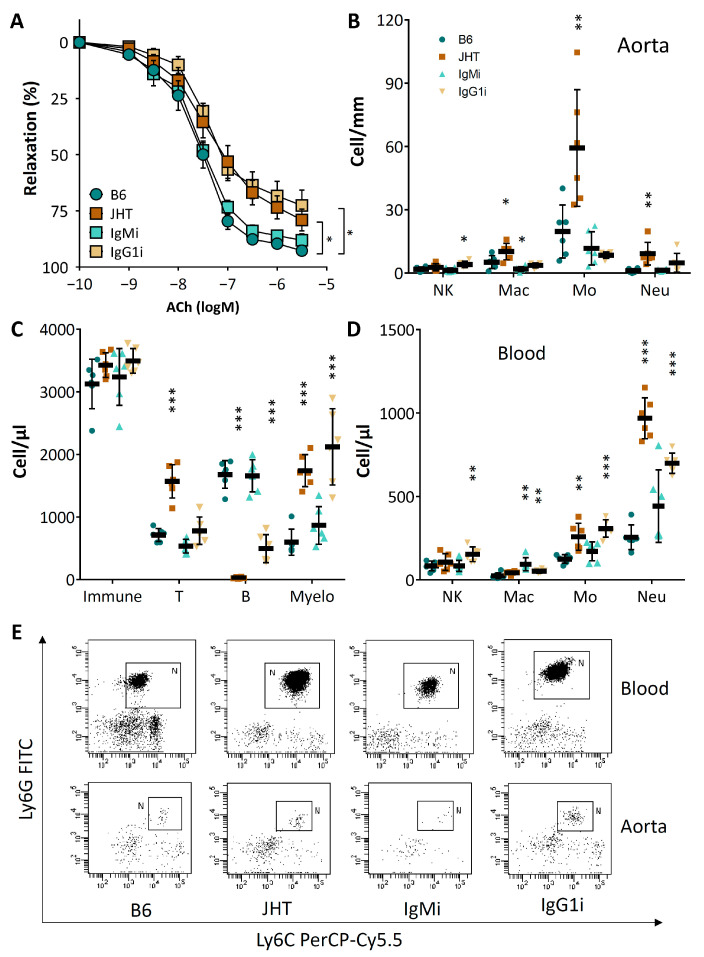
Vascular dysfunction in JHT and IgG1i mice is associated with neutrophilia. Vascular function (**A**) and immune cell profiles in aorta (**B**) and blood (**C**,**D**) were analyzed with samples from 7 week-old wild-type C57BL/6J, JHT, IgMi and IgG1i mice, respectively. Panel (**E**) shows flow cytometry of neutrophils in blood and aorta, respectively. Symbols in panel A represent mean ± SEM; n = 12. The horizontal lines in the scatter dot plots in panels B-D represent mean ± SD. * *p* < 0.05, ** *p* < 0.01, *** *p* < 0.001, compared with wild-type mice (B6); two-way ANOVA.

The vasodilator function of the aortae from the IgG1i mice was reduced to a similar extent as that from the JHT mice (Figure 4A). By contrast, the vascular function of the IgMi mice remained normal (Figure 4A). Both JHT and the IgG1i mice showed increased numbers of neutrophils in the vascular tissue as well as in the peripheral blood (Figure 4B–E).

**Figure 5:**
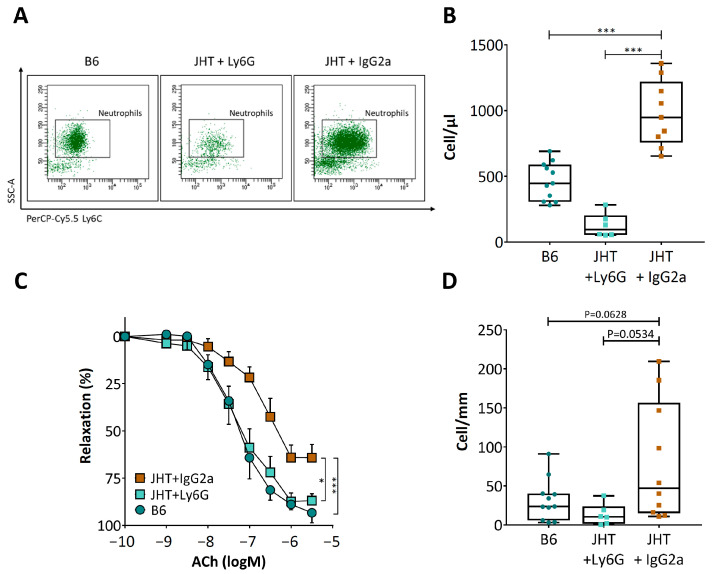
Neutrophil depletion restores vascular function in JHT mice. Ten-week-old JHT mice were treated intraperitoneally with anti-Ly6G antibody to deplete neutrophils. IgG2a served as isotope control. 36 h after antibody injection, neutrophil numbers were quantified in the circulating blood (**A**,**B**) and aorta (including aortic PVAT) with FACS. Vascular function was studied using a wire myograph system (**C**). The boxes in panels B and D represent the interquartile range (IQR) which contains data between the 25th and 75th percentiles. The whiskers represent the minimum and maximum values. The horizontal lines within the boxes are the medians (**B**,**D**). Symbols in panel C represent mean ± SEM; n = 9. * *p* < 0.05, *** *p* < 0.001; two-way ANOVA.

To deplete their neutrophils, the JHT mice were treated with a single intraperitoneal injection of a neutrophil-specific anti-Ly6G antibody. IgG2a served as an isotype control. As shown in Figure 5A,B, anti-Ly6G treatment markedly reduced the neutrophil numbers. Importantly, the depletion of neutrophils by anti-Ly6G led to a complete normalization of vasodilator function (Figure 5C), which was associated with a reduced neutrophil number in the aortic tissues (Figure 5D).

### 3.4. Vascular Dysfunction in B Cell-Deficient Mice Is Improved by B-1 Cell Transfer

**Figure 6:**
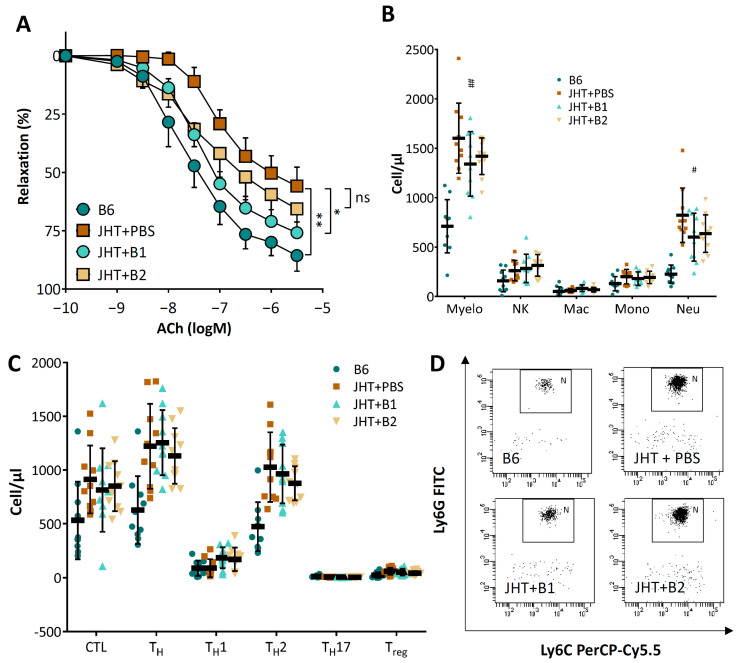
Adoptive B cell transfer improves vascular function in JHT mice. Eight-week-old JHT mice received 5 × 10^6^ B-1 or B-2 cells isolated from wild-type mice. Control JHT mice received the same volume of PBS. Two weeks after the adoptive B cell transfer, aortae were isolated for vascular function studies (**A**) and blood for immune cell profiling by FACS analyses (**B**–**D**). Symbols in panel A represent mean ± SEM; n = 8. The horizontal lines in the scatter dot plots in panels B and C represent mean ± SD. * *p* < 0.05, ** *p* < 0.01, compared with B6 mice; ^#^ *p* < 0.05, ^##^ *p* < 0.01, compared with JHT+PBS mice; two-way ANOVA.

To assess the role of the B-1 and B-2 cells, adoptive transfer experiments were performed in the B cell-deficient mice. The B cells were isolated from the 8 week-old wild-type mice and the B cell-deficient JHT mice received 5 × 10^6^ B-1 or B-2 cells, respectively [30]. Two weeks after B cell transfer, the aortae were isolated from the JHT-recipient mice for analyses of vascular function. As shown in Figure 6A, B-1 cell transfer significantly improved the vasodilator response of the JHT mice, whereas B-2 cells had only a minor effect. Interestingly, B-1 cell transfer in the JHT mice also reduced the numbers of circulating neutrophils, with no significant effect on T cell counts (Figure 6B–D).

## 4. Discussion

In the present study, we report the hitherto undescribed role of B lymphocytes in regulating vascular function. This seems to be a constitutive function of B cells under physiological conditions; an activation process is not required for this function of B cells. In our study, the mice were at a young age and not exposed to any known risk factors. This is a major difference with previous studies performed under pathological conditions, where B cells may lose their vasoprotective effects and become harmful.

A number of elegant studies have shown the roles of B cells in cardiometabolic pathologies. In diet-induced obesity, B cells can be activated by self-antigens, such as some intracellular proteins that are exposed due to hypoxia and apoptosis. Activated B cells promote insulin resistance by activating T cells and by producing pathogenic IgG antibodies [1]. In atherosclerosis models, hypercholesterinaemia and oxidative stress lead to the generation of oxidation-specific epitopes, on the surface of apoptotic cells and oxidized LDL (oxLDL) molecules [38]. B-1 cells produce specific natural IgM antibodies blocking oxLDL uptake, foam cell formation, and promoting apoptotic cell clearance [2,39,40]. By contrast, oxLDL-specific IgG antibodies produced by B-2 cells promote atherogenesis by activating macrophages, although they may also exhibit protective neutralizing capacities [2,39,40]. In angiotensin II-induced hypertension, B cells are activated by angiotensin II type 1 receptor stimulation [22].

In contrast to these studies, the mice in the present experiment were young, slim and without hyperlipidaemia or angiotensin II treatment. The lack of B lymphocyte alone leads to massive endothelial dysfunction (Figure 1), indicating that B cells play a crucial role in maintaining vascular homeostasis under physiological conditions. No blood pressure changes were observed in the JHT mice at the age of 17 weeks (Figure S1). Endothelial dysfunction is an early event; it precedes and predicts cardiovascular diseases [41]. However, an endothelial dysfunction does not necessarily translate immediately into blood pressure elevation [42]. It would be interesting to find out in future studies whether these mice are more prone to developing hypertension under challenging conditions, such as aging, high-fat diet-feeding or angiotensin II treatment.

Reduced NO production and enhanced oxidative stress are likely to be the molecular mechanisms underlying the observed endothelial dysfunction (Figure 1 and Figure 2). The reduced eNOS expression could be a result of TNF-α stimulation. TNF-α is known to down-regulate eNOS expression by destabilizing eNOS mRNA [43,44]. The increased concentration of neopterin in the circulating blood indicates not only an increased production of reactive oxygen but also cellular immune activation [45,46,47,48].

B cell-deficiency in mice caused an inflammatory response, characterized as increased numbers of T lymphocytes and neutrophils. The number of circulating neutrophils in the JHT mice was more than double that of the wild-type control (Figure 3). Importantly, neutrophil depletion with anit-Ly6G in the JHT mice led to a complete normalization of vascular function (Figure 5), indicating that the vascular dysfunction in B cell-deficient animals was mediated by neutrophilia. This conclusion is further supported by the results from the IgG1i mice (Figure 4) and the data from the B cell transfer experiments (Figure 6). The two mouse lines with neutrophilia (JHT and IgG1i) demonstrated vascular dysfunction, whereas the vascular function in the mice with normal neutrophil numbers (IgMi) remained completely normal. Furthermore, B-1 cell transfer in JHT mice improved vascular function, which was associated with a reduction in the neutrophil number. It is notable that the adoptive transfer experiments were performed with B-1 cells isolated from young and healthy wild-type mice not exposed to any risk factors. This suggests that the effects of B cells on neutrophils are conferred by naive B-1 cells without any stimulation/activation, i.e., it is a physiological function of these cells. Interestingly, B-1 cell transfer in the JHT mice had no effect on T cell number (Figure 6), suggesting that B-1 cells primarily regulate neutrophils, whereas the increased T cell number observed in the JHT mice (Figure 3) is likely to have been a secondary, compensatory response to B cell-deficiency. The fact that B-1 cell transfer improved vascular function without changing the number of T cells (Figure 6) indicates that T cells are not causally involved in the vascular dysfunction induced by B cell-deficiency.

The results from the B cell transfer experiments further indicate that B-1 cells are the B cell subset maintaining vascular function by regulating neutrophil number. Neutrophils are generated in the bone marrow. When profiling the immune cells in the bone marrow of the JHT mice, we found increased numbers of mature but also immature neutrophils (Supplementary Figure S7). These data suggest that B-1 cells control neutrophil haematopoiesis in the bone marrow. The mechanisms underlying the ways in which B cells regulate neutrophil haematopoiesis are still unknown and beyond the scope of this study. It has been reported previously that the frequency and activation status of neutrophils are significantly increased in spleens of lupus-prone mice when B cell maturation antigen (BCMA) is absent [49]. BCMA is the receptor for B cell activating factor (BAFF) and is critical for B cell survival in the bone marrow. Another study has shown that B cells can mobilize monocytes from the bone marrow into the blood by production of the chemokine Ccl7 [50]. Thus, evidence exists that B cells can regulate myelomonocytic cells. The principal regulator of neutrophil hematopoiesis is granulocyte colony stimulating factor (G-CSF), while IL-6 and granulocyte-macrophage colony-stimulating factor (GM-CSF) stimulate granulopoiesis as well [51,52]. However, the serum levels of G-CSF, GM-CSF and IL-6 were not different between the JHT and wild-type mice (Figure 2E and Figure S8). A recent study discovered that neutrophil hematopoiesis in the bone marrow is normally limited by orexin and a reduction of orexin production leads to neutrophilia [53]. Interestingly, the serum orexin levels in the JHT mice were significantly reduced relative to the wild-type mice (Supplementary Figure S8). Whether this is the mechanism responsible for neutrophilia caused by B cell-deficiency needs to be investigated in future studies.

Theoretically, the effects of B lymphocytes can be mediated by (i) antibody production; (ii) antigen presentation; and/or (iii) cytokine production. Because the IgMi mice (with a normal number of B cells but without the secretion of antibodies) demonstrated normal vascular function and the IgG1i mice (with B cells and IgG antibody only) demonstrated vascular dysfunction, it is postulated that the protective role of B cells in maintaining vascular function is mediated by B cells via cell–cell interaction or cytokine production, but not by antibody secretion. A possibility that cannot be excluded at this time, however, is that the IgG antibodies produced by the IgG1i mice overcame the protective role of the B cells. This may be an explanation for the vascular dysfunction in the IgG1i mice. The vasoprotective effects of B cells themselves, however, are likely to be antibody-independent, as seen in the IgMi mice.

The present study is also the first to demonstrate the role of neutrophils in regulating vasomotor function. Recent studies have implicated neutrophils in the progression of cardiovascular diseases, including atherosclerosis, thrombosis, acute coronary syndromes, heart failure and stroke [54,55]. The mechanisms underlying the effects of neutrophils in these processes are complex [54,55]. Upon stimulation, neutrophils release reactive oxygen species and granular proteins, including myeloperoxidase, matrix metalloproteinases, cathelicidin antimicrobial peptide, azurocidin 1, cathepsin G and elastase 2, resulting in monocyte recruitment and macrophage activation [54,55]. In addition, neutrophils form neutrophil extracellular traps (NETs) by releasing decondensed chromatin [56]. NET formation in inflamed blood vessels is known to generate a scaffold that promotes thrombosis, exacerbates damage in ischemia/reperfusion injury, and creates neo-antigens favoring autoimmunity [56]. Nevertheless, the effects of neutrophils on vasodilator function have not been reported so far.

The co-culture of endothelial cells with neutrophils leads to the enhanced expression of TNF-α, IL-6, IL-1β and intercellular adhesion molecule-1 (ICAM-1) (Figure S9), indicating that neutrophil contact can lead to endothelial activation and inflammation. It is believed that endothelial activation promotes immune cell recruitment and subsequent atherogenesis [57]. In addition to this traditional “inside-out” paradigm, recent studies have provided evidence supporting a new “outside-in” signaling concept [57]. The adventitia and PVAT contain far more inflammatory cells than the intima. In the case of PVAT inflammation, the vasa vasorum can function as channels for inflammatory cell trafficking, enabling immune cell migration from the PVAT to the intima [57,58]. In addition, small molecules from the PVAT can act directly on vascular cells, because there is no anatomic barrier between PVAT and the vascular wall [57]. We found evidence of neutrophil infiltration in the aorta and aortic PVAT of the JHT mice (Figure 3). Elevated levels of neutrophil proteins, including myeloperoxidase, neutrophil elastase and matrix metalloproteinase 9, were detected in the aortae or aortic PVAT of the JHT mice (Figures S4–S6). These neutrophil proteins may cause vascular inflammation and damage through the above-mentioned mechanisms.

The clinical relevance of B lymphocytes in cardiovascular disease was demonstrated in a recent study [59]. A network-driven integrative analysis has identified B cell-related genes as key drivers in coronary heart disease (CHD) [40,59]. Among the top 20 key drivers, 8 genes are directly related to B cell function or B cell receptor signaling, indicating that B cell dysregulation may be a major factor in atherosclerotic CHD [59].

## 5. Conclusions

The present study uncovers the novel function of B lymphocytes in maintaining vascular homeostasis under physiological conditions. The lack of B cells leads to vascular dysfunction by causing neutrophilia. Thus, B cell dysfunction may represent a crucial mechanism in cardiovascular disease.

## Data Availability

The data presented in this study are available on request from the corresponding author.

## Supplementary Materials

The following are available online at https://www.mdpi.com/article/10.3390/biomedicines9111686/s1, Figure S1: B cell-deficient JHT mice have normal blood pressure at the age of 17 weeks. Figure S2: B cell-deficiency leads to downregulation of eNOS without changes in the expression of nNOS or iNOS. Figure S3: B cell-deficiency leads to downregulation of SOD2 without changes in the expression of other redox genes. Figure S4: Increased levels of neutrophil protein myeloperoxidase (MPO) in the aorta and aortic PVAT of B cell-deficient JHT mice. Figure S5: Increased levels of neutrophil protein neutrophil elastase (NE) in aortic PVAT of B cell-deficient JHT mice. Figure S6: Increased levels of neutrophil protein metalloproteinase-9 (MMP9) in the aorta of B cell-deficient JHT mice. Figure S7: B cell-deficiency leads to enhanced neutrophil generation in the bone marrow. Figure S8: Effects of B cell-deficiency on factors of neutrophil hematopoiesis in the blood. Figure S9: Neutrophil contact leads to endothelial activation and inflammation. Figure S10: Downregulation of eNOS protein expression in JHT mice (whole blots for Figure 1E). Figure S11: Downregulation of SOD2 protein expression in JHT mice (whole blots for Figure 2C).

## Availability of Data and Material

Additional results are available online as supplementary data. Raw data and experimental materials used in this study are available upon reasonable request to the corresponding author.
