# Supplementary material for: B Lymphocyte-Deficiency in Mice Causes Vascular Dysfunction by Inducing Neutrophilia
Source: Biomedicines. 2021 Nov 14;9(11):1686. doi: 10.3390/biomedicines9111686 (PMC8615852; doi:10.3390/biomedicines9111686)
Supplement: Supplementary file 1 [file biomedicines-09-01686-s001.zip › biomedicines-1369958-supplementary.pdf]

Methods

Flow cytometric analyses

Cells were stained for 1 h with following commercial antibodies: CD45, CD19, CD3e, CD4, CD8, CD1d, CD5, CD11b, CD23, CD25, CD127, Gr1, F4/80, NK1.1, Ly6C, Ly6G, CXCR3 and CCR6 antibodies were obtained from BD Biosciences. Purified Rat Anti-Mouse CD16/CD32 (Mouse BD Fc Block™) (2.4G2) was from BD Biosciences. CD34, ckit, Gr1, Sca-1 and TER-119 antibodies were from eBiosciences. Streptavidin-APC-Cy7 were from BioLegend. LIVE/DEAD™ Fixable Near-IR Dead Cell Stain Kit, for 633 or 635 nm excitation, was from Thermo Fisher Scientific.

For blood samples, erythrocyte lysing was performed after staining by adding 1 ml of 1xBD PharmLyse™ lysing Buffer to 150 µl blood and incubation for 8 min at room temperature in the dark and washing three times with 1 ml FACS buffer (300 g, 6 min and 4 °C). Cells were suspended in FACS buffer and directly transferred into FACS tubes.

Aortas were separated from perivascular adipose tissue (PVAT) and shred vigorously with a razor blade before transferring the aorta tissue into 1 ml RPMI (Thermo Fisher Scientific) supplemented with 1 mg/ml liberase (Roche Diagnostics) and the PVAT into 1 ml HBSS supplemented with 200 U/ml collagenase 1 (Thermo Fisher Scientific). Samples were incubated at 37 °C and 500 g for 20 min in the dark. Enzyme digestion was stopped with 1 ml FACS buffer and cells were mechanically pressed through 70-µm cell strainers. Cells were harvested by centrifugation (300 g, 6 min and 4 °C) and kept in PBS until staining.

Bone marrow samples were obtained by flushing the lumina of hip and hind leg bones with RPMI supplemented with FCS using a 23G syringe. Cells were filtered through 70-µm cell strainers and centrifuged at 900 g and 4 °C for 5 min. Supernatant was discarded and cells were suspended in 2 ml 1xBD PharmLyse™ lysing Buffer. After incubation for 5 min in the dark, the lysing buffer was removed by centrifugation (900 g, 5 min and 4 °C). Cells were kept on FACS buffer until staining.

Single-cell suspensions were treated with Fc-block and washed. Cells were stained for 1 h with commercial antibodies. Dilutions were as recommended by the vendor. Samples were analyzed using a BD FACS CANTO II flow cytometer (Becton Dickinson) and FACSDiva Sofware 7.0 (Becton Dickinson), respectively.

T cell subsets were gated as T$_{h}$1: CD45^{+}, CD90.2^{+}, CD4^{+}, CD183^{+}, CD196^{-}; T$_{h}$2: CD45^{+}, CD90.2^{+}, CD4^{+}, CD183^{-}, CD196^{-}; T$_{h}$17: CD45^{+}, CD90.2^{+}, CD4^{+}, CD183^{-}, CD196^{+}; T$_{reg}$: CD45^{+}, CD90.2^{+}, CD4^{+}, CD25^{+}, CD127^{-}. B cell subsets were gated as B2: CD45^{+}, CD19^{+}, B220^{+}, CD23+; B1a: CD45^{+}, CD19^{+}, B220^{+}, CD23^{-}, CD5^{+}, CD1d^{-}; B1b: CD45^{+}, CD19^{+}, B220^{+}, CD23^{-}, CD5^{-}, CD1d^{+}; B$_{reg}$: CD45^{+}, CD19^{+}, B220^{+}, CD5^{+}, CD1d^{+}. Myelomonocytic cells were gated as NK cells: CD45^{+}, CD3e^{-}, CD11b^{+}, NK1.1^{+}; neutrophil granulocytes: CD45^{+}, CD3e^{-}, CD11b^{+}, NK1.1^{-}, F4/80^{-}, Ly6C^{+}, Ly6G^{+}; monocytes: CD45^{+}, CD3e^{-}, CD11b^{+}, NK1.1^{-}, F4/80^{+}, Ly6C^{-}, SSC-A$^{lo/hi}$; macrophages: CD45^{+}, CD3e^{-}, CD11b^{+}, NK1.1^{-}, F4/80^{+}, Ly6C^{-}, SSC-A^{neg}. Bone marrow cells were gated as HSC: CD3^{-}, CD4^{-}, CD8^{-}, CD5^{-}, B220^{-}, Gr1^{-}, Ter119^{-}, CD127^{-}, ckit^{+}, Sca-1^{+}; MEP: CD3^{-}, CD4^{-}, CD8^{-}, CD5^{-}, B220^{-}, Gr1^{-}, Ter119^{-}, CD127^{-}, ckit^{+}, Sca-1^{-}, CD16/32^{-}, CD34^{-}; CMP: CD3^{-}, CD4^{-}, CD8^{-}, CD5^{-}, B220^{-}, Gr1^{-}, Ter119^{-}, CD127^{-}, ckit^{+}, Sca-1^{-}, CD16/32^{-}, CD34^{+}; GMP: CD3^{-}, CD4^{-}, CD8^{-}, CD5^{-}, B220^{-}, Gr1^{-}, Ter119^{-}, CD127^{-}, ckit^{+}, Sca-1^{-}, CD16/32^{+}, CD34^{+}.

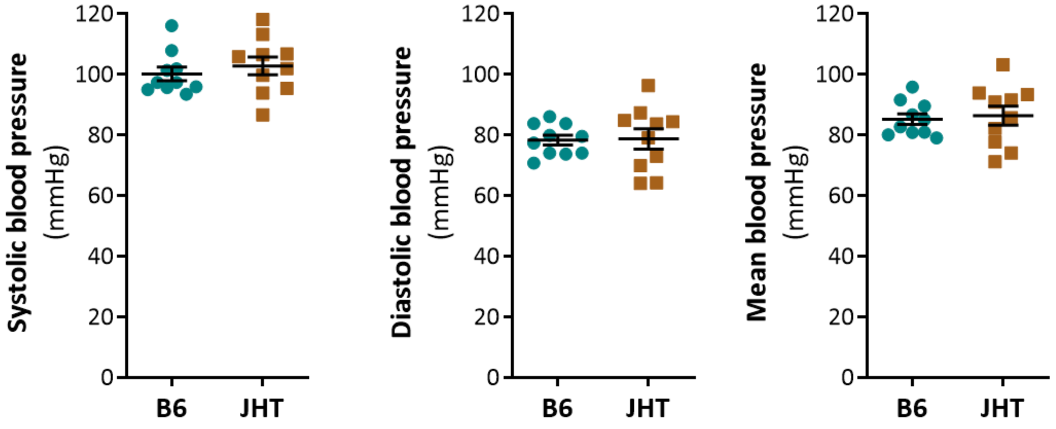

Figure S1. B cell-deficient JHT mice have normal blood pressure at the age of 17 weeks. Blood pressure was measured non-invasively in wild-type C57BL/6J and JHT mice at the age of 17 weeks using the CODA system (Kent Scientific). Data are presented as mean ± SEM. No statistically significant differences were found between B6 and JHT mice.

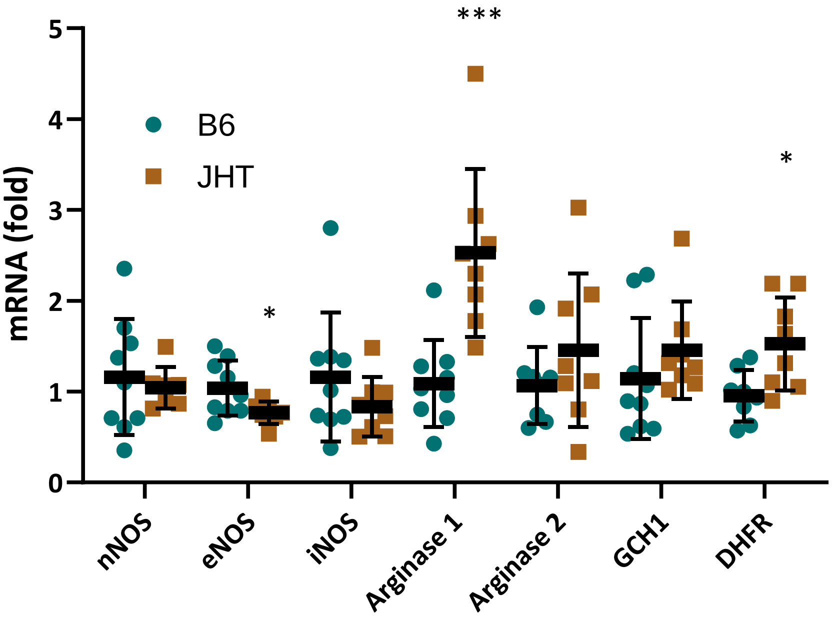

Figure S2. B cell-deficiency leads to downregulation of eNOS without changes in the expression of nNOS or iNOS. Total RNA was isolated from 17-week-old mice and mRNA expression analyzed with qPCR. Horizontal lines in the scatter dot plots represent mean ± SD. **p* < 0.05, ****p* < 0.001, compared with B6 mice. nNOS, neuronal NO synthase; eNOS, endothelial NO synthase; iNOS, inducible NO synthase. GCH1, guanosine triphosphate cyclohydrolase I; DHFR, dihydrofolate reductase.

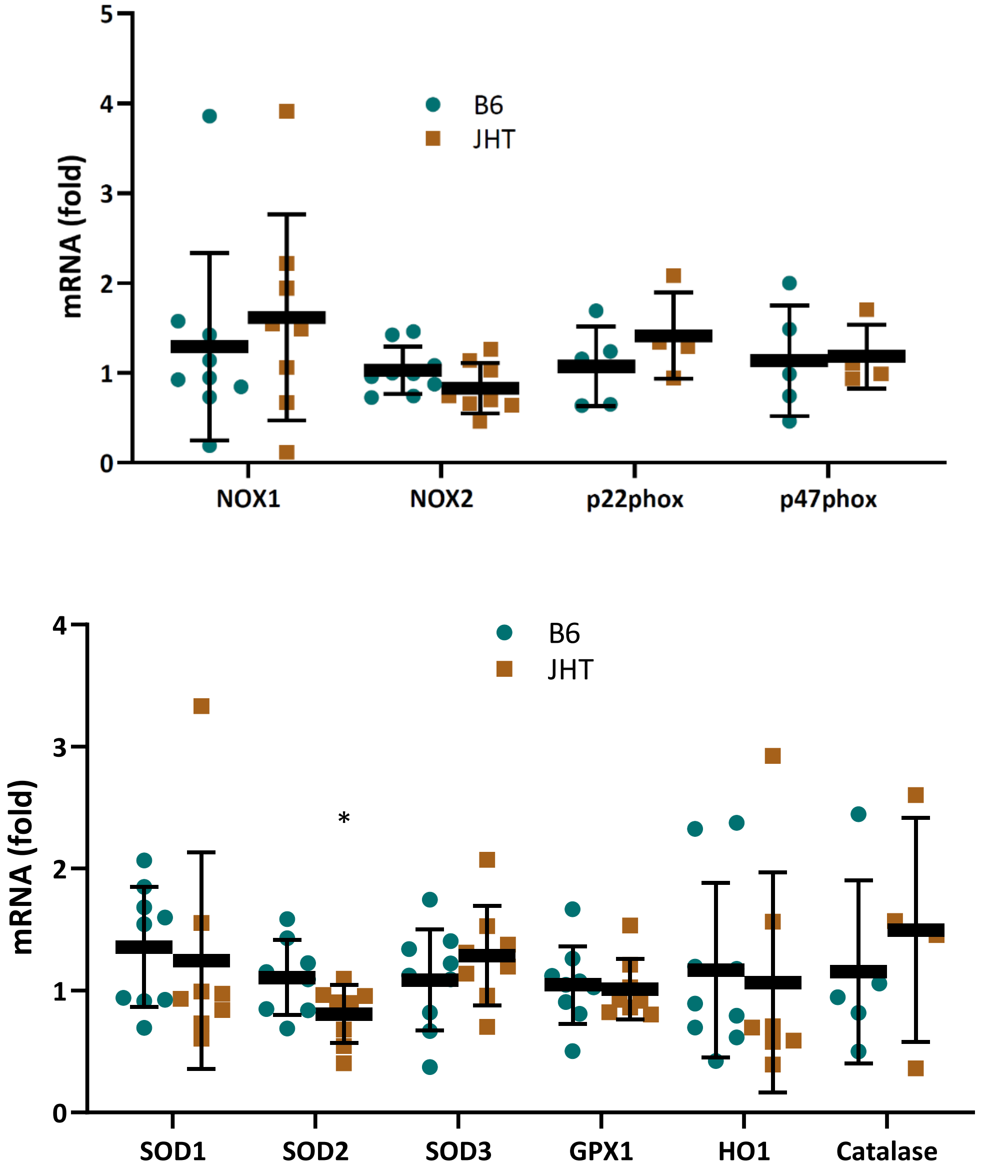

Figure S3. B cell-deficiency leads to downregulation of SOD2 without changes in the expression of other redox genes. Total RNA was isolated from 17-week-old mice and mRNA expression analyzed with qPCR. Horizontal lines in the scatter dot plots represent mean ± SD. *$p < 0.05$, compared with B6 mice. SOD, superoxide dismutase; GPX, glutathione peroxidase; HO, heme oxygenase; NOX, NADPH oxidase.

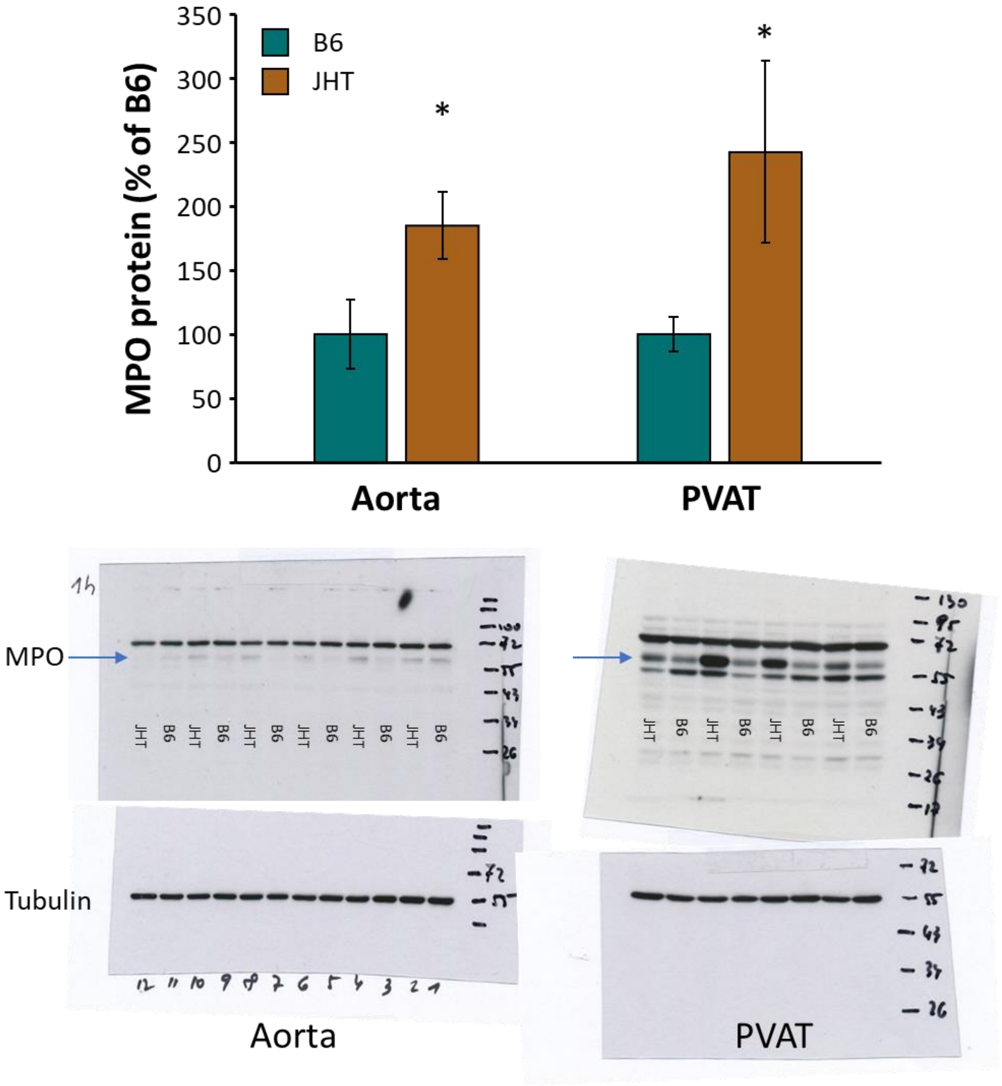

Figure S4. Increased levels of neutrophil protein myeloperoxidase (MPO) in the aorta and aortic PVAT of B cell-deficient JHT mice. Total protein was isolated from aorta and PVAT of 17-week-old mice, and Western blot analyses were performed using a rabbit monoclonal antibody against MPO (Abcam #ab208670; predicted band size 83 kDa, observed band size 59 kDa). β-tubulin was used as internal control. Data are presented as mean ± SEM. **p* < 0.05, compared with wild-type mice (B6), unpaired *t*-test.

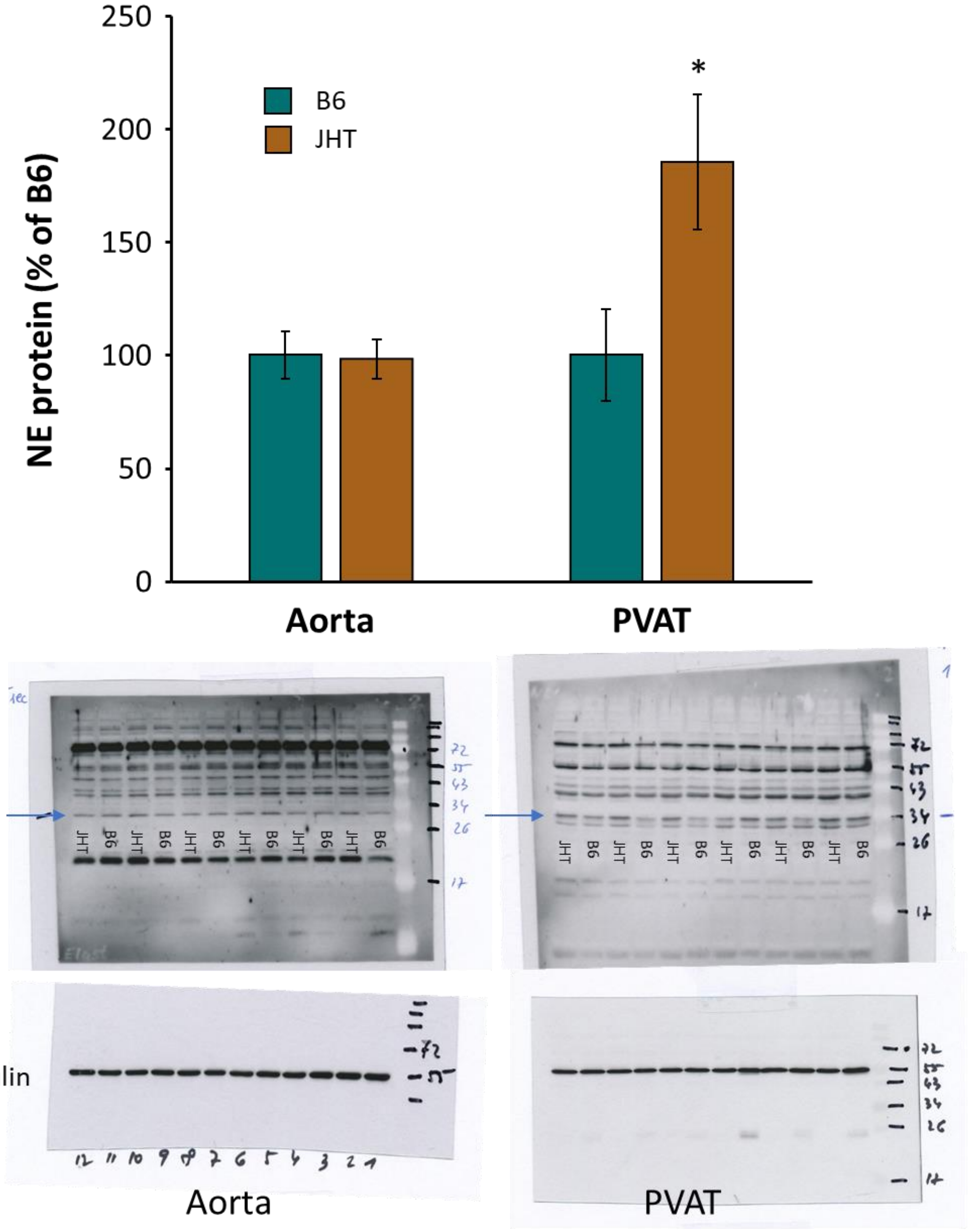

Figure S5. Increased levels of neutrophil protein neutrophil elastase (NE) in aortic PVAT of B cell-deficient JHT mice. Total protein was isolated from aorta and PVAT of 17-week-old mice, and Western blot analyses were performed using a rabbit monoclonal antibody against NE (Abcam #ab21595; 29 kDa). β-tubulin was used as internal control. Data are presented as mean ± SEM. *$p < 0.05$, compared with wild-type mice (B6), unpaired t-test.

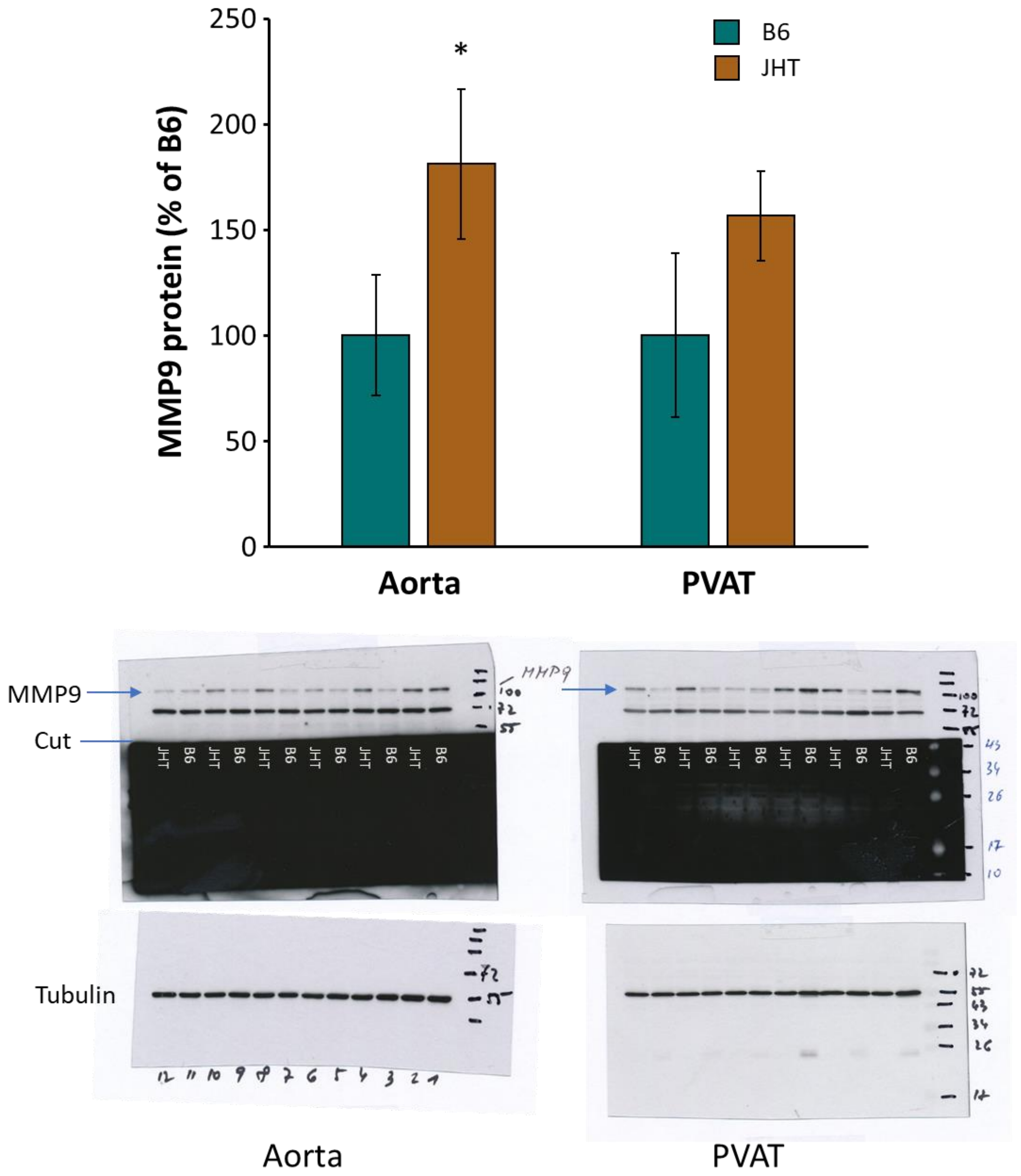

Figure S6. Increased levels of neutrophil protein metalloproteinase-9 (MMP9) in the aorta of B cell-deficient JHT mice. Total protein was isolated from aorta and PVAT of 17-week-old mice, and Western blot analyses were performed. The membrane was cut into two parts with the upper part stained using a rabbit monoclonal antibody against MMP9 (Abcam #ab228402). β-tubulin was used as internal control. Data are presented as mean ± SEM. *$p < 0.05$, compared with wild-type mice (B6), unpaired *t*-test.

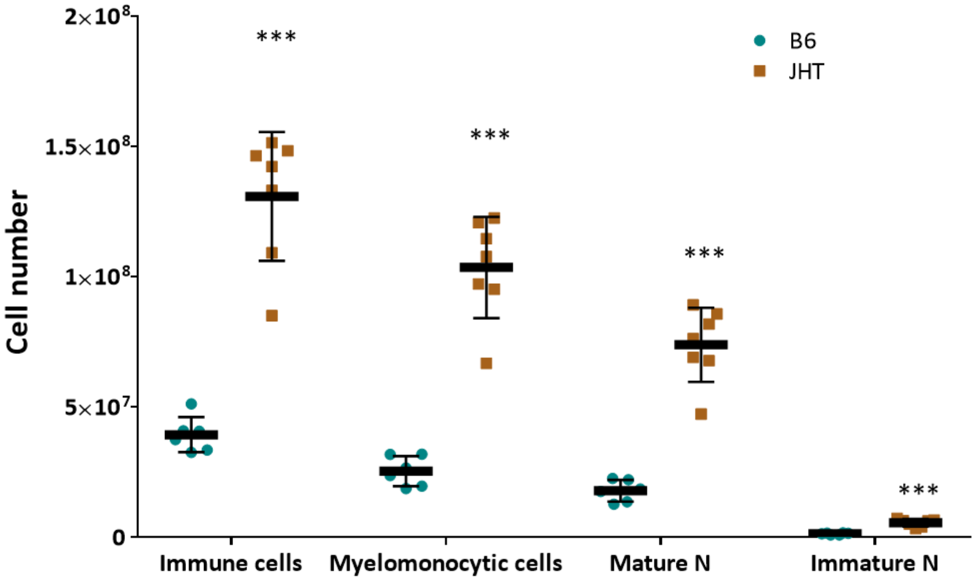

Figure S7. B cell-deficiency leads to enhanced neutrophil generation in the bone marrow. Immune cells were isolated from bone marrow of 17-week-old mice were analyzed with fluorescence-activated cell sorting (FACS). The horizontal lines in the scatter dot plots represent mean ± SD. *$p < 0.05$, **$p < 0.01$, compared with B6. N, neutrophils.

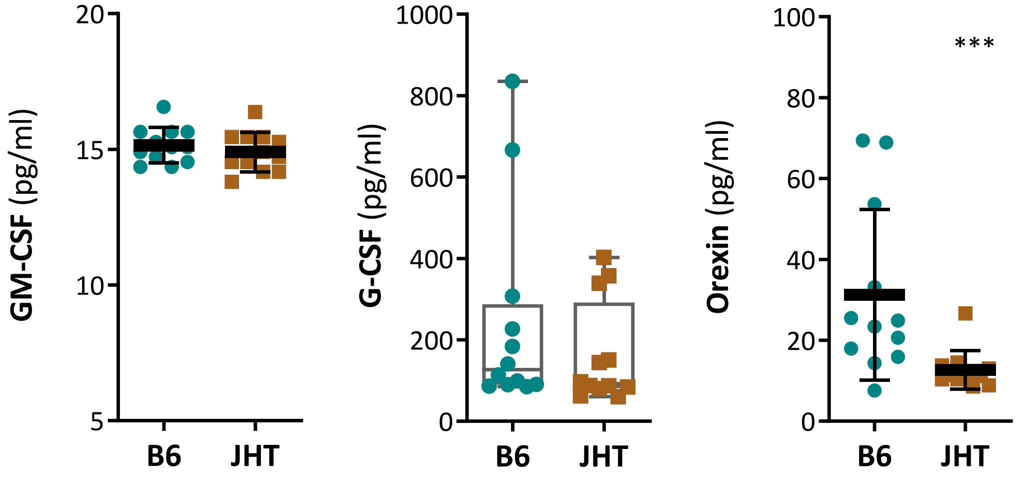

Figure S8. Effects of B cell-deficiency on factors of neutrophil hematopoiesis in the blood. Granulocyte-macrophage colony-stimulating factor (GM-CSF), granulocyte colony-stimulating factor (G-CSF) and orexin were measured in serum samples from 17-week-old mice. The horizontal lines in the scatter dot plots represent mean ± SD. The boxes in middle panel represent the interquartile range (IQR) which contains data between the 25th and 75th percentiles. The whiskers represent minimum and maximum values. The horizontal lines within the boxes are the medians. ***$p < 0.001$, compared with B6.

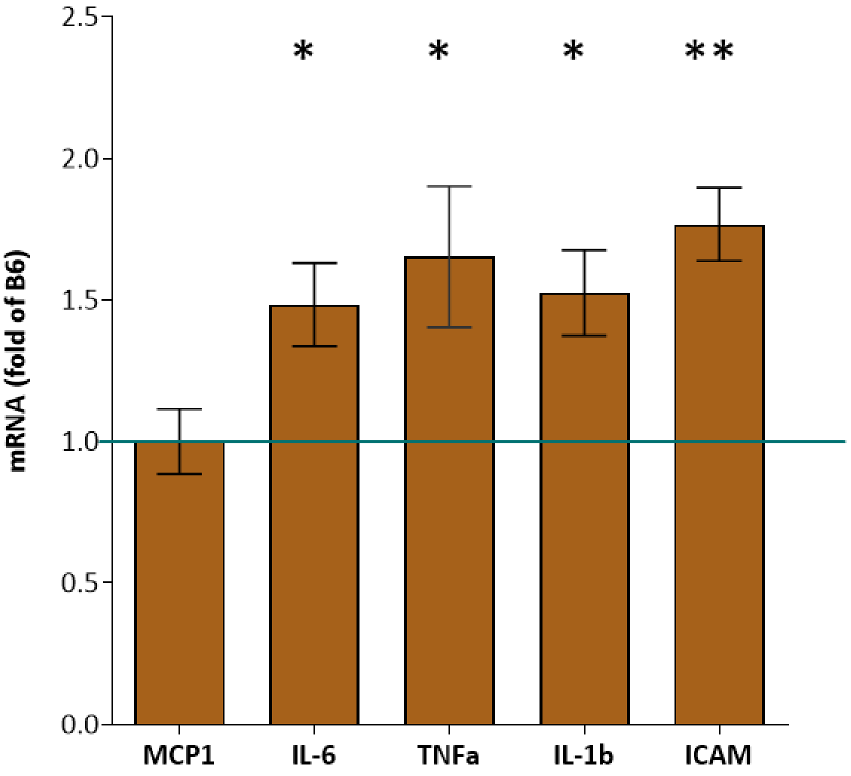

Figure S9. Neutrophil contact leads to endothelial activation and inflammation. Human EA.hy 926 endothelial cells were grown on 24-well plates till confluency. Then, mouse neutrophils isolated from the bone marrow were added to the endothelial cells at a density of 500,000 neutrophils/well. After a co-culture for 6 hours, the mouse neutrophils were removed, and the remaining human endothelial cells washed. Then, total RNA was isolated from the endothelial cells, and qPCR performed using primers for human MCP-1, IL-6, TNF-α, IL-1β and ICAM-1. Data are shown as mean ± SEM. *$p < 0.05$, **$p < 0.01$, compared to B6 mice (blue line). MCP-1, monocyte chemotactic protein 1; ICAM-1, intercellular adhesion molecule-1.

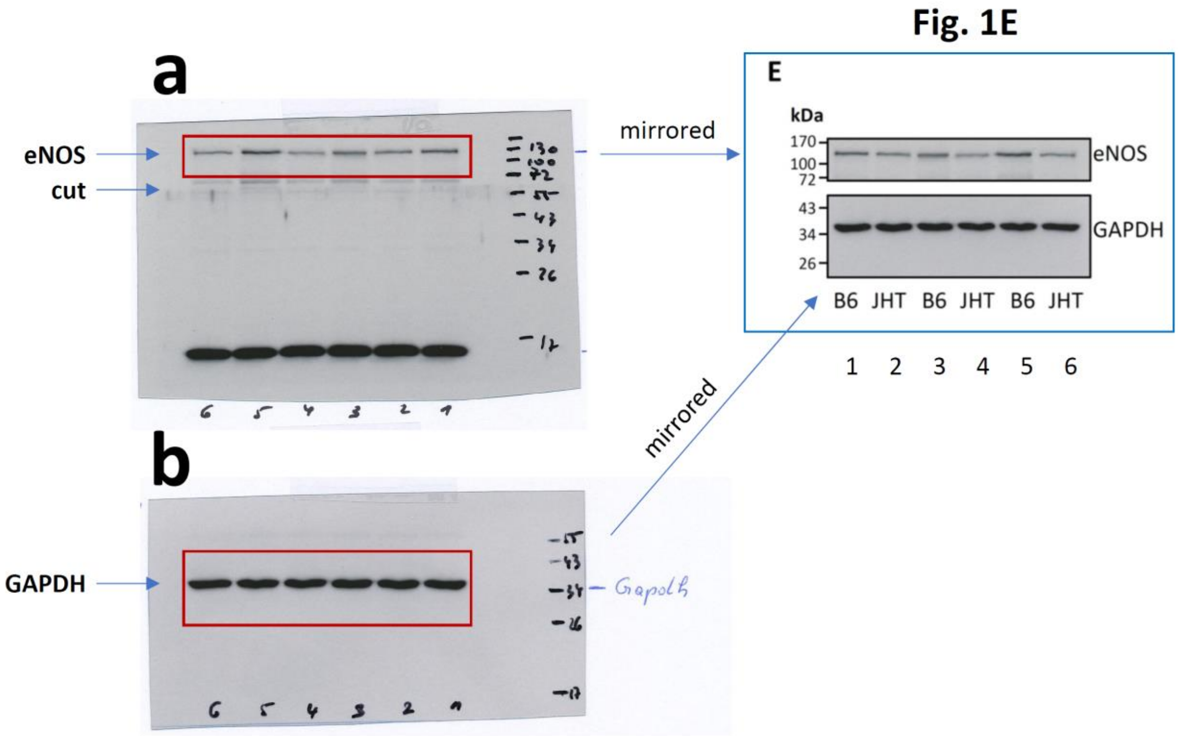

Figure S10. Downregulation of eNOS protein expression in JHT mice (whole blots for Figure. 1E). Protein samples were isolated from aorta of 17-week-old wild-type B6 or B cell-deficient JHT mice, respectively. The protein samples were separated on Bis-Tris gels and transferred to nitrocellulose membranes. (**a**) One membrane was cut into two parts, with the upper part being immunoblotted with an antibody against eNOS and the lower part against SOD1.(**b**) Another membrane was blotted with an antibody against GADPH. The red boxes indicate regions of interest for the main figure (Figure. 1E). For a better understanding, mirrored images of the original blots were used in Figure. 1E.

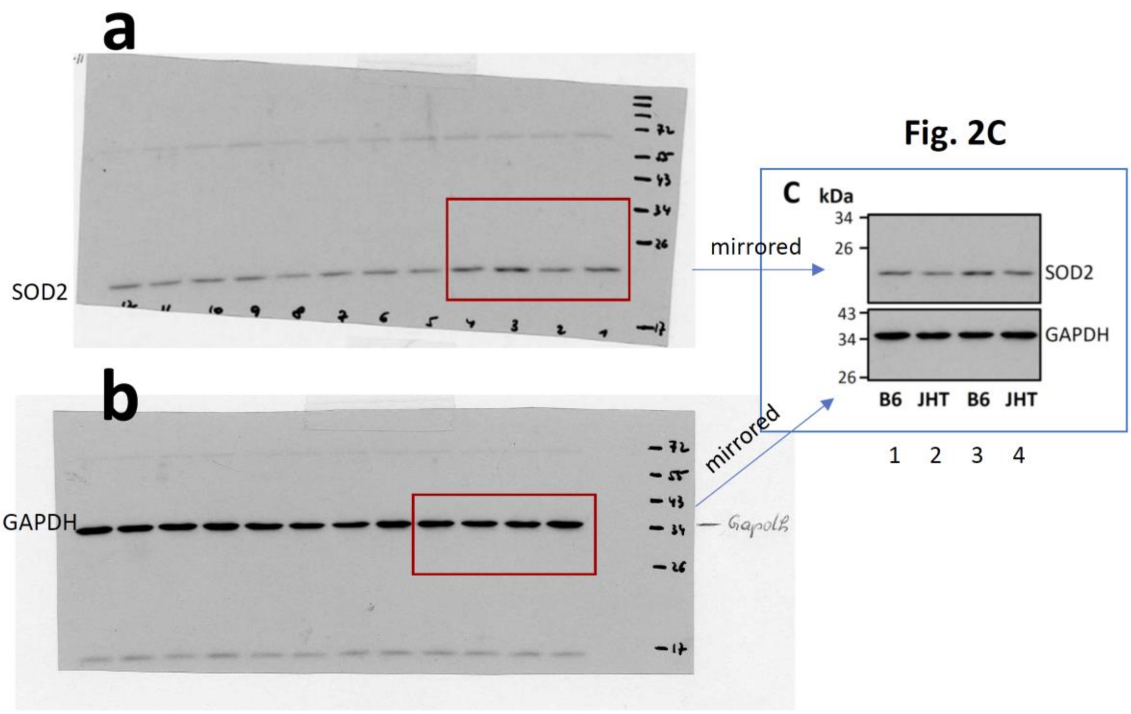

Figure S11. Downregulation of SOD2 protein expression in JHT mice (whole blots for Figure. 2C). Protein samples were isolated from aorta of 17-week-old wild-type B6 or B cell-deficient JHT mice, respectively. The protein samples were separated on Bis-Tris gels and transferred to nitrocellulose membranes. (**a**) One membrane was immunoblotted with an antibody against SOD2. (**b**) Another membrane was blotted with an antibody against GADPH. The red boxes indicate regions of interest for the main figure (Figure. 2C). For a better understanding, mirrored images of the original blots were used in Figure. 2C.
